# Naming and Necessity: Sherborn’s Context in the 19^th^ Century

**DOI:** 10.3897/zookeys.550.7399

**Published:** 2016-01-07

**Authors:** Gordon McOuat

**Affiliations:** 1 University of King’s College, Halifax, NS, CANADA

## Abstract

By the late 19^th^ Century, storms plaguing early Victorian systematics and nomenclature seemed to have abated. Vociferous disputes over radical renaming, the world-shaking clash of all-encompassing procrustean systems, struggles over centres of authority, and the issues of language and meaning had now been settled by the institution of a stable imperial museum and its catalogues, a set of rules for the naming of zoological objects, and a new professional class of zoologists. Yet, for all that tranquillity, the disputes simmered below the surface, re-emerging as bitter struggles over synonyms, trinomials, the subspecies category, the looming issues of the philosophy of scientific language, and the aggressive new American style of field biology – all pressed in upon the received practice of naming and classifying organisms and the threat of anarchy. In the midst rose an index. This paper will explore the context of CD Sherborn’s *Index Animalium* and those looming problems and issues which a laborious and comprehensive “index of nature” was meant to solve.

## Editor’s note

This paper is a transcription of the talk presented by Professor McOuat in the symposium *Anchoring Biodiversity Information: from Sherborn to the 21^st^ century and beyond*, 28 October 2011, Natural History Museum, London. It is an exciting read about an important topic for this volume – it sets the historical and philosophical context for Sherborn’s contribution to nomenclature and taxonomy clearly and vibrantly. It has a number of key messages on the relationships between names (dubbing) and meanings (taxonomy), on the struggle between establishing nomenclature tied to rules (codes) or to specimens (the type concept and museum catalogues). These issues were intensely addressed in the early and mid 19^th^ century and Sherborn’s magnum opus played a foundational role in establishing the systems we now use for all biology, not just zoology. Nonetheless many taxonomists today continue to befuddle these relationships, often through lack of knowledge of the long history of the discussions. I felt it was critical that this history is included in this volume, because it adds a different and necessary perspective on Sherborn’s context and influence. Although we were not successful in getting Gordon McOuat to send his written text for the volume, I have decided to publish this as a transcript, with minor edits for flow and a few images for expanded context, as the talk is in the public domain and its presentation was fully funded by the symposium organisers. The paper should thus be read as a transcript only.

## Talk and slides

http://backdoorbroadcasting.net/2011/10/gordon-mcouat-sherborn%E2%80%99s-context-cataloguing-nature/

## Early Victorian recognition of the value of names

Although he worked in the late 19^th^ Century, Sherborn’s context starts with the very earliest groundwork for modern taxonomy, systematics and nomenclatural practice in the early 19^th^ Century. This time included the origins of well-known disputes, of ruckuses in early Victorian biology, some of which are still with us today. Understanding these origins helps understand the issues in Victorian times and today.

Early Victorians knew the value of names, often couching the discussion in monetised terms. Sir William Kirby, in his Foundational Address of the Zoological Club of the Linnean Society, 1823, expressed the value that a name brings:

### Nomina si pereunt, perit et cognitio rerum

“Names are the foundation of knowledge: and unless they have a ‘a name’ as well as a ‘local habitation’ with us, the zoological treasures that we so highly prize might almost as well have been left to perish in their native deserts or forests, as have grown mouldy in our drawers or repositories. But when once an animal subject is named and described, it becomes a possession for ever, and the value of every individual specimen of it, even in a mercantile view, is enhanced.”

This is matched by the words of the radical anatomist, Robert Grant in his presentation to the Parliamentary Commission on the Affairs of the British Museum in 1835–1836:

‘An object may not be the value of a farthing until it is identified and properly named. Its value may be raised to 30, 40 or 50 guineas once it is named, even though it has not gained an ounce.’

Both Kirby and Grant expressed the value of names at a time when there was turmoil in the process of giving names, and there was a process being born to establish stability and an anchor. There was a radical new club in the Linnean Society of London that harboured those who aimed to break the hold of Linnaeus over systematics and meaning. They aimed to introduce new ideas, imported, for example from France, to break the hold of the Linnaean world system. This is where Kirby made his presentation. Robert Grant, as a radical who called for the overthrow of all received systems, was himself a Lamarckian, an evolutionist and political radical before Darwin. These are presented in ‘reform-bill Britain’ where democratic forces threatened Tory privileges, much as the Occupy movement attempted at St Paul’s, or as we see in the current challenges to the existing political and economic systems.

**Figure 1.**
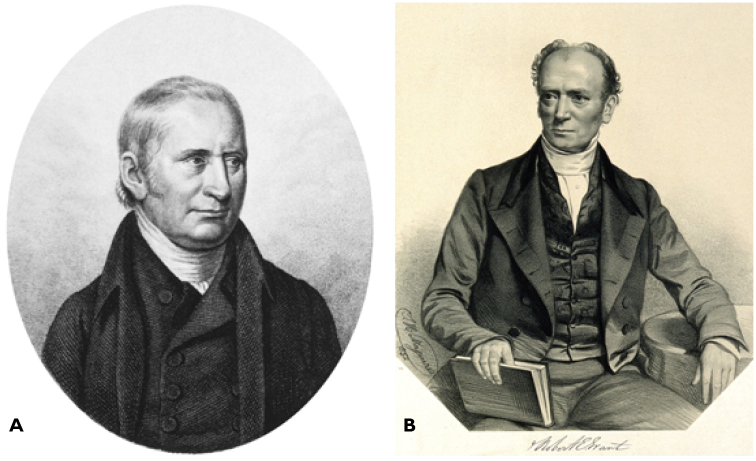
**A** Sir William Kirby, date and age uncertain **B** Robert Grant in 1852, aged 59.

## The Stricklandian Code – the first attempt at an international code governing language in any science.

Any conference on nomenclature – its problems and its history – must harken back to the pioneering document, the founding creed of nomenclatural rules, the Code of Zoological Nomenclature drafted in 1842 by Hugh Edwin Strickland (1811–1853) under the patronage of the British Association for the Advancement of Science.

Strickland’s committee was a veritable who’s who of British natural history: John Stevens Henslow, Jennings, William Ogleby, JO Westwood, Richard Owen, Charles Darwin, William Yarl, WE Shuckard and GR Waterhouse. The committee convened its meetings in Darwin’s house, as he still lived in London at the time. Here is an early draft of Strickland’s rules with Darwin and Ogilby’s comments on what should be changed and what should be kept (courtesy of Cambridge University Library): I cannot over emphasise the importance of these rules as a founding document. They are the first attempt at an international code governing language in any science. Any modern code, whether botanical or zoological, can trace its direct ancestry to this code. Many of the structures of modern codes, and many might say some of the problems, and zoological nomenclature in particular, can be traced directly to this code and its rules.

There are some important peculiarities of this document and its inheritance. We should unpack it a bit and give some grounding for Sherborn and his monumental project. The Stricklandian Code starts with a series of paragraphs with a very detailed account of the philosophy of language.

**Figure 2.**
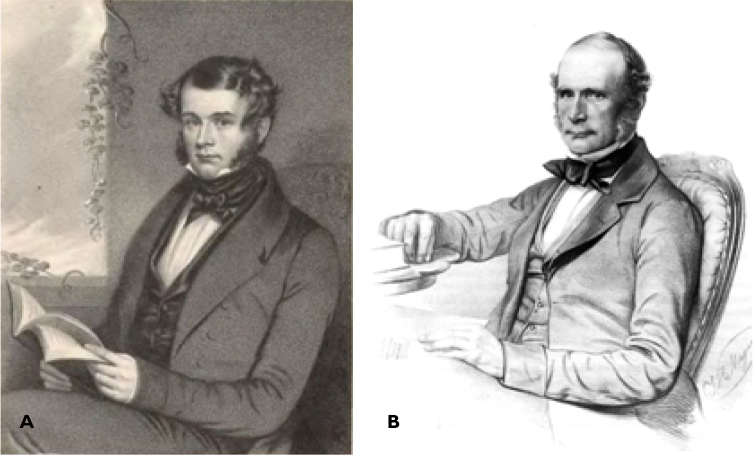
Hugh Edwin Strickland **A** age 26 **B** aged 42, when he died.

**Figure 3.**
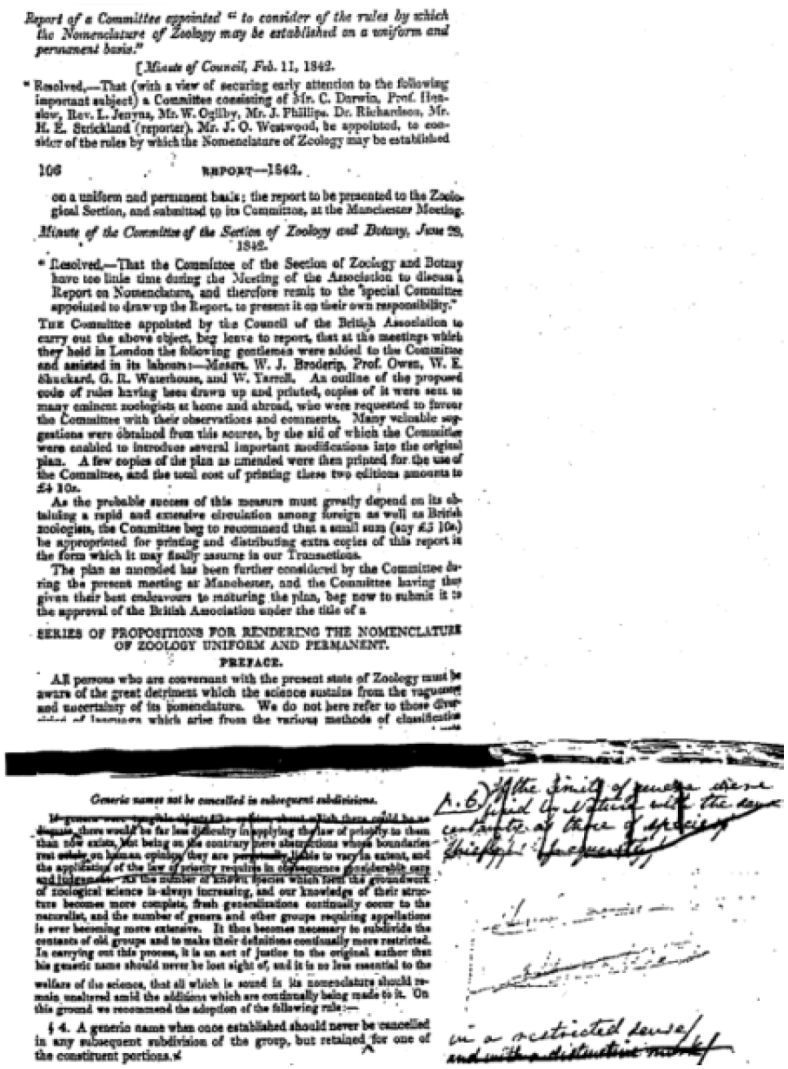
Early draft of the Stricklandian Code with handwritten comments by Darwin and Ogilby.

## Language and meaning – dubbing not definitions

The system of naming and reference were in contention in Britain at this very time; followers of William Whewell had entirely different understanding of how things were named from the followers of John Locke. The Stricklandian Committee held a Lockian view of the meaning of meaning, as so remarkably espoused in these paragraphs.

Strickland himself had written numerously and voluminously on the notion of language, on the meaning and use of names. Strickland wrote:

‘Words are only conventional signs. This should be enough to check those who are constantly trying to subvert the language of zoology. Names do not capture essences; they are not definitions. So how do they get authority and reference? By first dubbing. Not by accurately capturing any meaning or sense, but rather that very first dubbing. These rules are about dubbing, and about disciplining that dubbing.’

**Figure 4.**
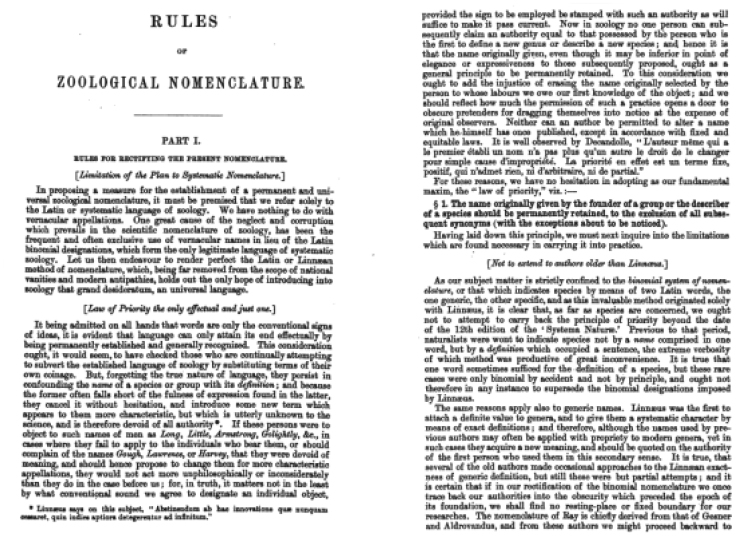
Stricklandian Code – discussion of the philosophy of language.

This is a remarkable start for a set of rules on zoological nomenclature. It is only understandable in the face of the radical attempts, all through the early 19^th^ century, and also today, to radically alter the words or names of things to match their place in scientific place and practice; to have names capture the reform and meaning of science.

Strickland had cut his teeth on fighting such radical attempts, and they were legion in the early 19^th^ century, to entirely reform the whole system zoology and to adjust the names of things to match that reform.

Strickland’s biggest enemy was Neville Wood, who was a popular writer, ornithologist, and eventually one of the leaders of alternative medicine in the late 19^th^ century. Neville Wood would write ‘It is essential for the improvement of ornithological science that names be frequently altered, for when a new system is proposed – and there are few who would advocate the Linnaean system now – new names must necessarily be introduced.’

New systems abounded. Anyone who is an historian of early 19^th^ century natural history knows there were bifurcating systems and quinarian systems and Cuvierians…all of them associating the new names that they were establishing to match their system. But for Strickland, names are arbitrary – they are dubbings that hold on to that reference irrespective of the meanings in the systems to which they belong. But, asked Strickland, if there is a first dubbing, where is it to occur? Somewhat contravening his own philosophy, Strickland gives an arbitrary date of the 12^th^ edition of Linnaeus’ *Systema Naturae* where he thinks we find the solidification of binomial (binominal) nomenclature. It is from that moment that the dubbing of names should begin. This is where Strickland introduces the law (now principle) of priority. The very first rule states “the name originally given by the founder of a group or the describer of a species should be permanently retained.”

Here the rules are giving rules for procedure and not for construction (or meanings) of the names themselves. The rest of the Code outlines where such descriptions can be found: published in certain received authoritative journals and books, and not in the popular press. All this was aimed at preventing amateurs from forming new names willy-nilly, removing the anchor and changing the very nature of zoological discourse. Thus, Strickland kept the issue of the meaning of names at bay. But notice how this brings up the issue of priority and genealogy.

## The overarching priority of priority

The emergence of the Stricklandian Code was not without its own controversy. The ‘British Association for the Advancement of Science Rules’ were not actually passed by that organisation. They were cleverly inserted by Strickland into the report of 1842, but they were not actually adopted by the BAAS because of enormous opposition to the first rules.

The strongest opposition came from John Edward Gray (1800–1875), the chief Keeper of Zoology at the British Museum (which was still in Bloomsbury), who kept the rules from being approved by the BAAS. He was adamant that the Stricklandian rules should not be established to control the nature of discourse in natural history. Why? Because he was, at the same time, establishing a different source for authority on naming and discourse for natural history. He was working on his own solution to systematic and nomenclatural anarchy, his own material anchor to the biodiversity problem. For Gray, the British Museum catalogues of types would establish names and reference and be the site of authority. Not some regulatory rules, but real concrete catalogues and type specimens that would solidify the names.

Interestingly this huge fight between rules and museums continued in to the middle of the 19^th^ century. Gray used the anarchy that seemed to exist in zoology as a way to lobby aristocratic trustees of the British Museum to publish the catalogues and establish them as the worldwide authorities of types and thus species. These began publication in the 1840s.

The huge fight had its short-term resolution, in a certain sense, in Darwin. His monograph on barnacles was the first to explicitly use the new Stricklandian rules, but also the first to use the new Gray catalogues. It was Darwin who tried to create a resolution of the rules from the committee of which he was a member, and the catalogues of types. This was an uneasy compromise that has not been a complete success – the controversy ran through the late 19^th^ century.

**Figure 5.**
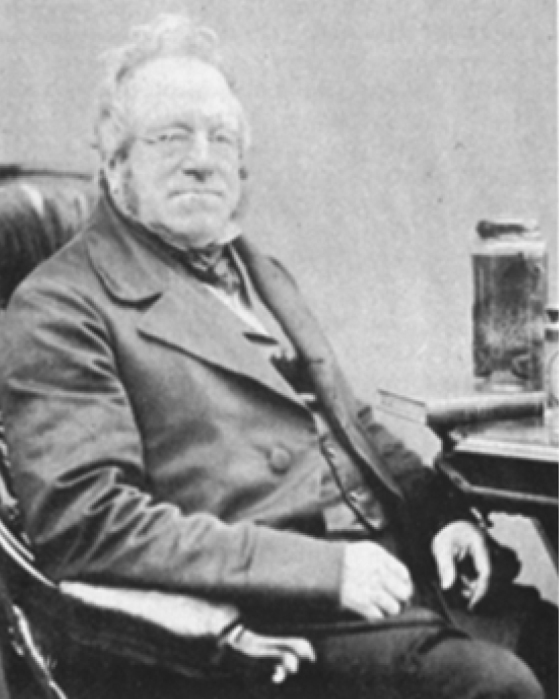
John Edward Gray, the chief Keeper of Zoology at the British Museum (Bloomsbury).

**Figure 6.**
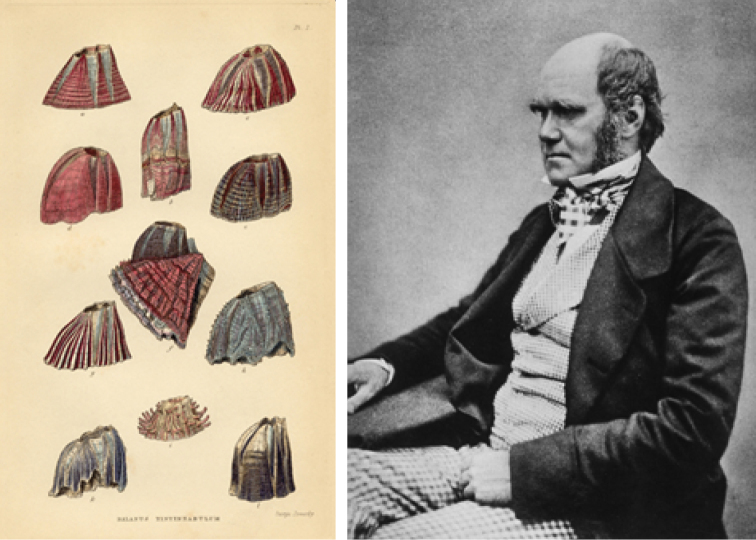
Charles Darwin and a plate from his work on barnacles.

**Figure 7.**
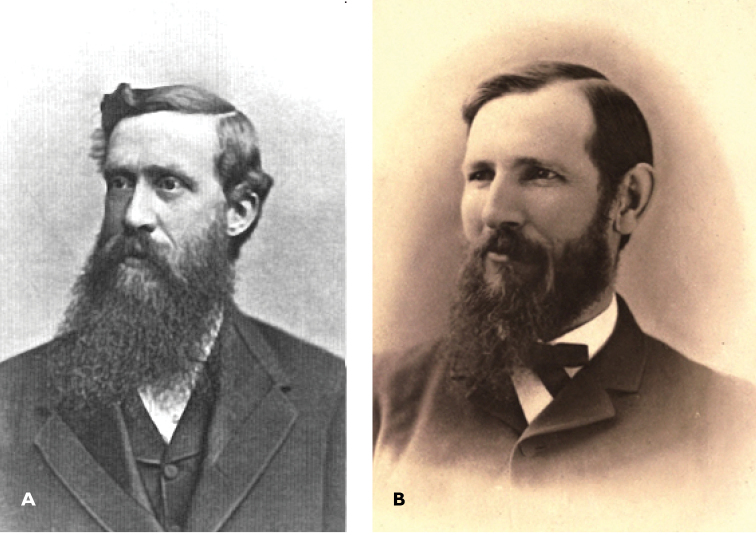
**A** Elliot Coues **B** Joel A. Allen

It was then in the new Natural History Museum in South Kensington where the next steps were taken in the great nomenclatural debate, and the seeds were sown for Sherborn’s great project. A great but controversial American zoologist, Elliot Coues (1842–1899) (Evenhuis this volume; Dickinson, this volume) happened to be visiting spiritualist sites throughout Europe. He and his partner in crime, Joel A. Allen (1838–1921), arrive at the Natural History Museum and advocated a new American way of doing field-based zoology, specifically ornithology, instead of the stodgy museum-based biology of the Old World. They set out an ornithological set of rules for nomenclature, which was supported by the American Ornithological Union.

The AOU rules were basically grounded on the Stricklandian rules except for one striking addition – the introduction of subspecies names, based on geographical distribution. Organisms would now be identified by a trinomial that would include the geographical location. All three parts would comprise the organism’s name. For the British this was an utter travesty from ignorant Americans that promised a return to anarchy, to use the phrasing from William Flower, the director of the Natural History Museum’s words. For the British, this was clearly mixing up, negating, the original Lockian perspective. It mixed up naming and meaning, violating all that had been achieved in establishing a system-free nomenclatural authority.

**Figure 8.**
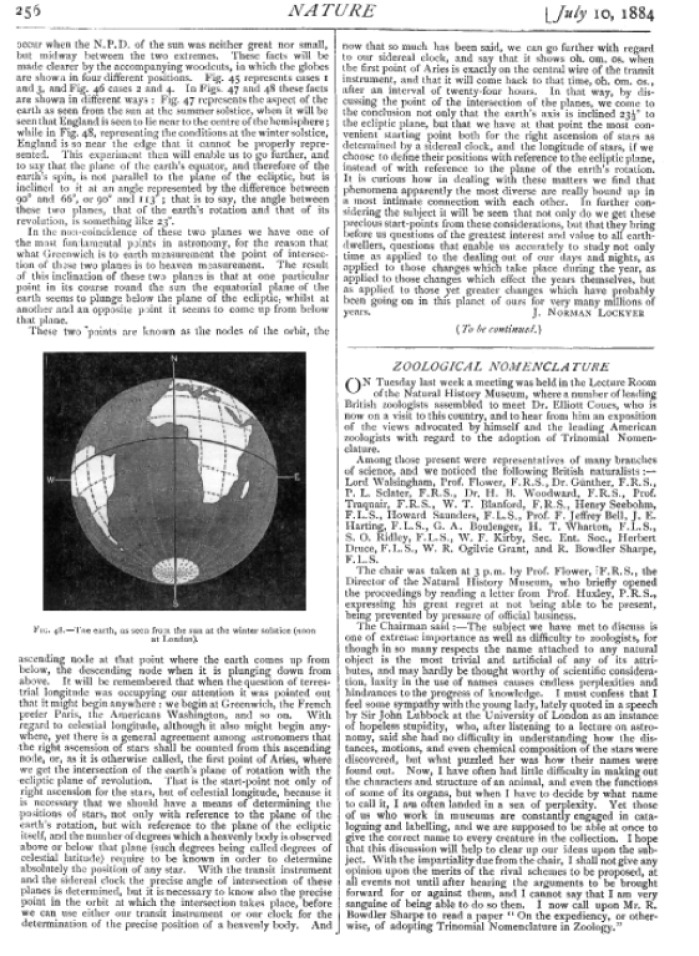
Report of the meeting discussing trinomial nomenclature.

Thus, on July 1^st^, 1884 Coues presented his new system of trinomial nomenclature in a meeting in the new Natural History Museum London. Every British zoologist of note was there – Schlater, Bolter, Guenther, Sharp. Huxley sent a note saying he couldn’t attend but give’em hell. All were there to give the upstart Americans, Coues and Allen, a piece of their minds and defend their rules and their museum. The verbatim report from *Nature* makes interesting reading from a philosophical standpoint, as all the debates from the early 19^th^ century are rehearsed in 1884 (Fig. 8). In fact, these arguments about the meaning of language, of dubbing, of authority, are rehearsed again and again subsequently, and perhaps still through the ICZN. Fears of anarchy are continually raised if there were to be a rejigging the ‘meaning of meaning’ for all of zoological nomenclature.

The British scientists argued that, by identifying location, trinomials were giving meaning within the name itself. This was liable to abuse, and would destabilise the system of authority so deeply established by rules and by the museum. Coues attempted to fight his corner but to no avail. The meeting was raucous and Coues was sent limping. The debate lasted long past that Tuesday 1^st^ July, carrying on for the rest of the year in the press and journals.

## Enter Sherborn and the *Index*

**Figure 9.**
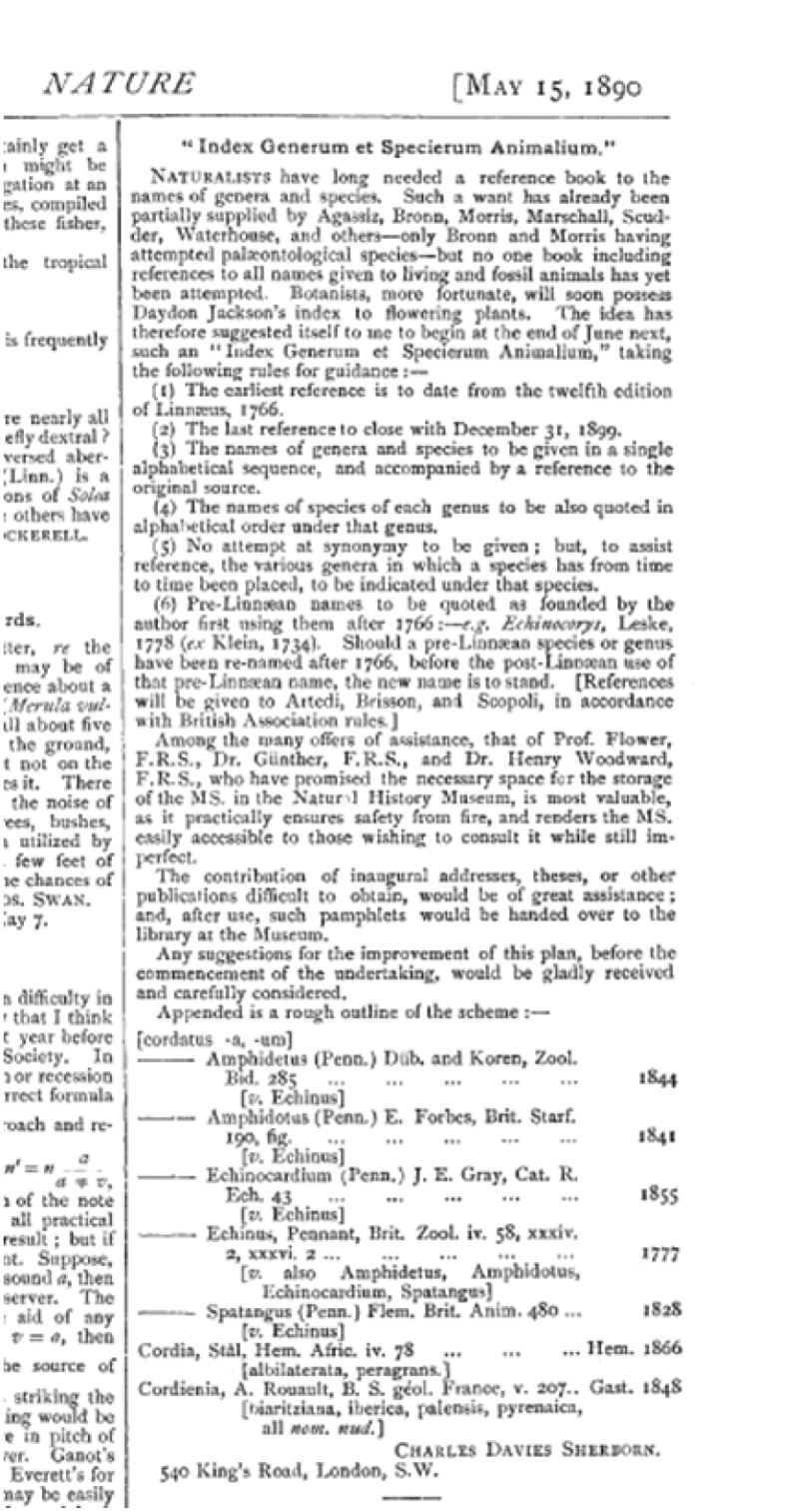
Announcement of Sherborn’s plans for his hugely ambitious project.

Recently employed by the geologist Thomas Rupert Jones in the British Museum (who might well have attended the raucous discussions on rules and meaning in names), was the 23 year-old Charles Davies Sherborn. He had already shown a predilection for indexing. We saw that Elliot Coues had tried to provoke him by saying only an inspired idiot could perform such a work. With the inspiration of Flower, Guenther, Slater and others, Sherborn published the announcement for the project of his great work in the May 1890 issue of *Nature* (Fig. 9). As stated in the announcement, the index was to be built on binomials (binominals) alone. He would constantly write that if something was a trinomial, it was not a name. The list was alphabetised by species, not genus. And the philosophical rule of priority, of first dubbing, now set to be from the 12^th^ edition of Linnaeus, was to apply. The index became a deciding foundation to the problematic first presented by an attempt to anchor zoological discourse in a philosophy of language. It was a method of grounding and dubbing. It wore its origins and philosophical genealogy proudly.

**Figure 10.**
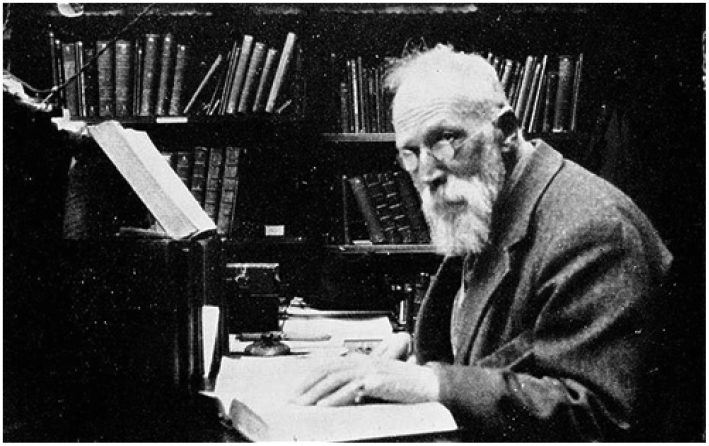
An iconographic picture of Sherborn in later years – staged, but revealing and taken at about the time of the final quote.

As Sherborn stated later in life, in a 1933 private letter to Vaughn, the head of the Scripps Institute in the United States,

“After all this work, there are only two rules that are any good: First - Priority, which dates from 1^st^ January 1758, and Second, that the first trivial is the type. If the generic diagnosis does not agree, then so much the worse for the genus, and it must be revised, unless the type is specifically mentioned. Them’s my sentiments.”

Sherborn continues,

“The International Zoological Committee is of little value as it meets only once in five years and then talks, but decides nothing. What we want is a Mussolini who can decide. Not a congress or a conference or such body who merely argue and make suggestions. I regard the first trivial name in a genus as the type unless it is otherwise fixed.”

Images are in the public domain through Wikipedia and Wikimedia Commons unless otherwise noted.

